# Health-related quality of life of individuals dealing with cancer in the Free State: A survey

**DOI:** 10.4102/sajp.v81i1.2094

**Published:** 2025-01-09

**Authors:** Roline Y. Barnes, Karen Bodenstein, Mariëtte Nel

**Affiliations:** 1 Department of Physiotherapy, Faculty of Health Sciences, University of the Free State, Bloemfontein, South Africa; 2 Department of Biostatistics, Faculty of Health Sciences, University of the Free State, Bloemfontein, South Africa

**Keywords:** FACT-G, HRQoL, cancer, physiotherapy, well-being

## Abstract

**Background:**

Cancer is the main cause of morbidity and mortality worldwide, and its symptoms can affect an individual’s life holistically.

**Objectives:**

Our study aimed to determine the health-related quality of life (HRQoL) of individuals dealing with cancer in the Free State, South Africa.

**Method:**

A descriptive, cross-sectional study design utilising the standardised Functional Assessment of Cancer Therapy – General (FACT-G) questionnaire as well as a self-developed sociodemographic and general health information questionnaire was used in this study. The study was conducted at the Universitas Annex Oncology Clinic.

**Results:**

A total of 507 participants were conveniently sampled. The median age was 53 years with 73.8% being female. The FACT-G’s overall score ranged from 11.7 to 108, with a median of 76.7 (interquartile range [IQR] 63–89.5). The Social or Family Well-Being subscale indicated the highest median of 22.2 and the Emotional Well-Being subscale the lowest of 18. The Physical Well-Being subscale found that 61.8% of individuals felt forced to spend time in bed and 53.4% experienced nausea. The Functional Well-Being subscale showed that 70.3% of individuals accepted their illness and 51.6% enjoyed their life.

**Conclusion:**

Our study highlights the importance of using a clinical assessment tool to determine the influence of cancer on the individual’s HRQoL. Involvement of the individual’s family and friends during management, as well as identifying the need for psychosocial support, is crucial for positively influencing HRQoL.

**Clinical implications:**

The FACT-G is a valuable tool in guiding physiotherapists and other healthcare professionals with patients’ cancer-related treatment experiences.

## Introduction

In Africa, approximately 2000 lives are lost daily because of cancer, which has become a notable public health challenge worldwide (Omotoso et al. 2023). Numerous factors, including ageing, unhealthy lifestyle choices and population growth, have contributed to the increased projection of 70% new cancer cases by 2030 (Jedy-Agba et al. 2020; Hamdi et al. 2021; World Health Organization [WHO] 2022). In South Africa, a person’s carcinogen exposure plays a big role in the development of cancer. This includes pesticides, industrial and consumer products and pollution. These factors cause unusual immune system responses, and a series of complications can arise that affect the patient’s health-related quality of life (HRQoL) and incur financial costs (Herbst 2017). Ageing is another major factor in cancer incidence and mortality, with most cases reported between 36 and 55 years (Hamdi et al. 2021). Despite the increasing mortality rate, cancer is not prioritised by stakeholders and policymakers in addressing the impact that the disease and its treatment has on individuals dealing with cancer, their families and communities (Hamdi et al. 2021).

Patient-centred HRQoL has become an important health outcome for clinical practice (Buiting & Olthuis 2020; De Boer et al. 2016; Zapatero et al. 2021; Zheng et al. 2021). Health-Related Quality of Life is defined as ‘how well a person functions in their life and his or her perceived well-being in physical, mental, and social domains of health’ (Karimi & Brazier 2016:646). Determining patients’ HRQoL and functional ability is essential for healthcare professionals to provide insight into patients’ emotional, physical, social or family and functional well-being (Buiting & Olthuis 2020). In addition, HRQoL identifies specific individual needs and perceptions that might influence treatment decisions facilitating person-centred care, focusing on survival and treatment-related experiences (Shahjalal et al. 2023). Determining HRQoL among individuals dealing with cancer throughout their treatment also aligns with the sustainable development goals (SDGs), particularly goal three, aimed at good health and well-being (Sustainable Development Goals 2024). Furthermore, understanding how HRQoL outcomes vary with sociodemographic and clinical factors may offer insight into clinical practice, health policy and individualised healthcare interventions (Shahjalal et al. 2023).

Cancer research is not limited to medical interventions as a form of treatment; it also extends to physiotherapy and the effects thereof on the HRQoL of patients (Sweegers et al. 2019). Although improvements in cancer treatment have led to a decline in death rates among cancer patients, these treatments are usually accompanied by side effects, which negatively affect the patient. Along with the side effects, treatment-related experiences can also impact the patient’s HRQoL long after therapy has concluded (CANSA 2022).

Two outcome measures are available to determine HRQoL of individuals dealing with cancer, the Functional Assessment of Cancer Therapy – General (FACT-G) questionnaire (FACT-G 2023) and the European Organisation for Research and Treatment of Cancer Quality-of-Life Questionnaire Core 30 (EORTC QLQ-C30) (Hand 2016; Luckett et al. 2011). Both these questionnaires have their merits; however, they cover different aspects of HRQoL despite also having a fair amount of overlap. A study conducted by Kemmler et al. (1999) indicated that neither of the two instruments can be replaced by the other, but the EORTC QLQ-C30 seemed to concentrate largely on the physical and functional aspects of quality of life, while the aim of our study was to include all the domains of an individual’s life. The FACT-G is commonly used in oncology research (Schurr et al. 2023) and several studies (Jabari, Nawajah & Jabareen 2022; Schurr et al. 2023) have used the FACT-G to study and determine the HRQoL in individuals dealing with cancer. Therefore, the FACT-G was chosen as measurement instrument for our study.

The FACT-G consists of four subscales, Social or Family Well-Being (SWB), Physical Well-Being (PWB), Functional Well-Being (FWB) and Emotional Well-Being (EWB). SWB refers to the entire family dynamics and functioning. This changes because of cancer, and the progression or recovery affects patients as well as their families and caregivers emotionally and psychologically. Understanding how psychosocial factors influence the entire course of cancer is becoming increasingly more important (Wang & Feng 2022). The PWB refers to the physical activities that are essential for the functional independence of a person (Schurr et al. 2023) and the ability to carry out activities and social tasks without physical limitations and bodily pain or fatigue (Capio, Cindy & Abernethy 2014). This is determined by self-reported symptoms, including a lack of energy, nausea, trouble with meeting the needs of the patient’s family, pain, side effects of the treatment, feeling ill and being forced to spend time in bed (Schurr et al. 2023). Functional Well-Being is a pivotal HRQoL indicator in individuals dealing with cancer and grades the capability of individuals to conduct basic tasks inside and outside their homes (Schurr et al. 2023). Sánchez, Ballesteros and Arnold (2011) describe EWB as a person’s ‘emotional state’, which includes individual’s emotional symptoms and how they cope with their illness.

Health-Related Quality of Life in individuals dealing with cancer influences the outcome of their treatment (Jansen van Rensburg, Maree & Casteleijn 2017) and impacts their functional independence (Schurr et al. 2023). Research on the HRQoL of individuals dealing with cancer has been conducted worldwide (Jabari et al. 2022; Schurr et al. 2023), as well as in a public hospital in Gauteng, South Africa (Jansen van Rensburg et al. 2017). The aim of our study was to determine the HRQoL of individuals dealing with cancer in the Free State, South Africa by using the FACT-G questionnaire.

## Research methods and design

A descriptive cross-sectional study design utilising the standardised, self-administered FACT-G questionnaire as well as a self-developed questionnaire to gather sociodemographic and general health information was used. The FACT-G is a well validated and reliable questionnaire (Costet et al. 2005; Sánchez et al. 2011). Costet et al. (2005) indicated satisfactory internal consistency of the global FACT-G scale (Cronbach’s alpha *α* = 0.9) and subscales (*α* > 0.75). The highest possible score for the PWB, SWB and FWB is 28, and for the EBW, is 24 (Brucker et al. 2005). The four subscales of the FACT-G questionnaire are scored with a 5-point Likert-type scale. The FACT-G consists of 27 items that evaluate HRQoL and is aimed at individuals dealing with cancer from the age of 18 years and older, based on the individual’s life experiences during the past 7 days. The scores range from zero to four, where four is the highest and zero is the lowest possible score per individual question. There are seven questions per subscale with some items required to be reversed scored. A total FACT-G score is calculated by combining all the subscales’ scores. The lowest possible total FACT-G score is zero, and the maximum score is 108 (Brucker et al. 2005). No normative data exist for disease, symptom- or condition-specific subscales, but higher scores for the different scales and subscales indicate a higher HRQoL (Benjamin, Jason & Lauren 2021).

Our study population consisted of individuals dealing with cancer admitted to the oncology wards or attending the oncology clinic at Universitas Annex in Bloemfontein. The monthly admissions at Universitas Annex range between 1000 and 1200 patients. Convenience sampling was used, and the recommended sample size of 341 individuals was calculated using the Raosoft sample size calculator (Raosoft 2004). Participants were included if they were 18 years and older, provided informed consent, were diagnosed with any type of cancer and were literate in either Afrikaans, English or Sesotho. A pilot study, following the same procedure as the main study, was conducted after ethical approval was obtained. Recruitment and data collection took place from February to April of 2023.

The information document was provided to each participant in their language of choice. Participation in our study was voluntary and participants who provided informed consent were handed questionnaires to complete. Confidentiality of the participants was ensured by numbering the questionnaires. Researcher assistants were available to assist the participants throughout the process.

After completion of the questionnaires, the authors transferred the data to a password-protected Excel spreadsheet and hard copies of the questionnaires were stored in a lockable safe. Data analyses were performed by the Department of Biostatistics, University of the Free State (UFS). Descriptive statistics were calculated for both numerical and categorical data.

### Ethical considerations

Ethical approval for our study was obtained from the Health Science Research Ethics Committee (HSREC) (UFS-HSD2022/1452/2911), Gatekeepers of the UFS and the Free State Department of Health. Participants were informed that they were allowed to withdraw from our study at any stage and that no risks were involved. Participants were informed that they would receive no remuneration to participate in our study, and if the study’s findings were to be published, it would be presented in group format and not participant specific. The study data were managed according to the *Protection of Personal Information Act 4 of 2013 (POPIA)*.

## Results

The total number of participants was 507, of whom 73.8% were female. The median age was 53 years. More than half (59.0%) who participated were human immunodeficiency virus (HIV) positive. Breast cancer (39.3%) was the most prevalent cancer, followed by cervical (16.96%) and prostate cancer (10.3%). It was noteworthy that 46.9% were unaware of the stage of their cancer and 63.5% were receiving chemotherapy during the time that our study was conducted. A summary of the main sociodemographic information of our study population can be viewed in Table 1.

**TABLE 1:** Summary of sociodemographic information (*N* = 507).

Variables	%
**Language (*n* = 507)**	
Sesotho	64.3
English	5.1
Afrikaans	21.3
**Marital status (*n* = 501)**	
Single, not married	23.8
In a relationship, not married	7.6
Married	38.3
Divorced	7.6
Widowed	15.0
**Educational level (*n* = 506)**	
No schooling	4.9
Primary school	17.0
Secondary school	56.3
Tertiary education	20.2
**Employment (*n* = 507)**	
Working full-time	13.8
Working part-time	4.7
Piece jobs	5.1
Unemployed	33.9
Pensioner	39.1
Self-employed	4.5
**Home (*n* = 490)**	
Own home or flat	77.3
Lives with friend or family member	22.7
**Type of housing (*n* = 501)**	
Brick house	85.4
Informal housing	13.0
**Electricity (*n* = 507)**	
Yes	95.3
No	4.7
**Water (*n* = 506)**	
Yes	90.3
No	9.7
**Living arrangement (*n* = 505)**	
Lives alone	6.3
Lives with 1 person	9.5
Lives with 2 persons	22.6
Lives with 3 or more persons	61.6

The total score on the FACT-G questionnaire ranged from a minimum of 11.7 to a maximum of 108, with a median of 76.7. Median and interquartile range values are reported as the numerical data were not normally distributed. The highest median illustrated (refer to Table 2) was from the SWB subscale (22.2), whereas the lowest median was from the EWB subscale (18). However, the mean values were also calculated to allow for comparison to other studies.

**TABLE 2:** Summary of Functional Assessment of Cancer Therapy – General scale scores (*N* = 507).

Variable	Median	Lower quartile	Upper quartile	Minimum	Maximum	Mean	Standard deviation
FACT-G total	76.7	63	89.5	11.7	108	74.6	19.0
SWB subscale score	22.2	17	25.7	1.0	28	20.9	6.1
FWB subscale score	20.0	15	26.0	0.0	28	19.2	7.2
PWB subscale score	19.0	13	24.0	0.0	28	17.8	7.2
EWB subscale score	18.0	13	22.0	0.0	24	16.7	6.2

Note: Higher scores indicate better quality of life.

EWB, Emotional Well-Being; FACT-G, Functional Assessment of Cancer Therapy – General; FWB, Functional Well-Being; PWB, Physical Well-Being; SWB, Social or Family Well-Being.

No association was found between age and the FACT-G total score (*p* = 0.3). When comparing the median total score of the FACT-G, individuals dealing with skin cancer experienced the lowest HRQoL (69) and those with brain cancer the highest (85). It is interesting to notice that 23 participants were unsure about which cancer they were dealing with (refer to Table 3).

**TABLE 3:** Functional Assessment of Cancer Therapy – General total scores for each type of cancer (*N* = 478).

Type of cancer	*n*	Median	Lower quartile	Upper quartile	Minimum	Maximum	Mean	Standard deviation
Breast	199	75.2	61.0	89.3	27.0	107.0	74.0	18.6
Prostate	52	74.3	55.1	93.8	11.7	105.6	72.9	22.0
Lung	22	75.0	52.5	84.0	36.5	98.0	70.9	19.6
Cervical	86	76.0	67.0	89.0	25.3	108.0	74.6	19.4
Colon	21	76.5	64.0	84.0	38.0	101.0	74.6	15.8
Skin	23	69.0	57.1	86.0	37.8	108.0	71.8	19.2
Throat	12	80.7	66.8	89.8	48.0	98.0	77.8	15.1
Brain	6	85.0	74.3	100.0	73.2	104.0	86.9	12.8
Lymph	20	81.3	70.6	94.2	24.0	107.0	80.9	19.2
Oesophagus	2	64.2	40.0	88.3	40.0	88.3	64.2	34.2
Bone	12	75.0	59.8	88.5	44.0	99.0	73.6	18.5

Note: Unsure (*n* = 23).

The subscale items for which more than 50% of participants indicated a ‘4’ (‘very much’) are highlighted in Table 4.

**TABLE 4:** Subscales of Functional Assessment of Cancer Therapy – General scale scores (*N* = 507).

Subscale items	*n*	Not at all	A little bit	Somewhat	Quite a bit	Very much	Frequency missing
**Physical well-being subscale results (%)**							
I have a lack of energy	505	16.4	16.8	14.7	25.7	26.3	2
I have nausea	498	8.6	7.2	12.3	18.5	53.4†	9
Because of my physical condition I have trouble meeting the needs of my family	498	23.5	14.3	11.5	15.3	35.5	9
I have pain	498	22.7	14.7	13.5	17.5	31.7	9
I am bothered by side effects of treatment	492	23.8	10.4	9.4	13.4	43.1	15
I feel ill	502	14.9	13.9	12.6	14.9	43.6	5
I am forced to spend time in bed	505	9.3	8.3	7.3	13.3	61.8†	2
**Emotional Well-Being subscale results (%)**							
I feel sad	502	14.3	11.2	15.9	17.3	41.2	5
I am satisfied with how I am coping with my illness	502	9.4	8.0	10.2	22.3	50.2†	5
I am losing hope in my fight against my illness	501	12.6	7.6	5.0	9.0	65.9†	6
I feel nervous	499	12.4	9.8	11.4	18.2	48.1	8
I worry about dying	502	19.1	6.4	7.4	10.4	56.8†	5
I worry that my condition will get worse	506	22.1	9.7	8.1	13.2	46.8	1
**Functional Well-Being subscale results (%)**							
I am able to work (include work at home)	506	14.8	14.6	16.4	19.2	35.0	1
My work (include work at home) is fulfilling	500	17.0	13.6	12.4	19.6	37.4	7
I am able to enjoy life	500	10.0	9.0	12.6	16.8	51.6†	7
I have accepted my illness	502	6.0	4.2	4.8	14.7	70.3†	5
I am sleeping well	503	14.5	12.9	13.1	18.7	40.8	4
I am enjoying the things I usually do for fun	507	16.2	12.6	12.4	16.0	42.8	0
I am content with the quality of my life right now	507	11.4	8.3	12.6	20.3	47.3	0
**Social or Family Well-Being subscale results (%)**							
I feel close to my friends	502	20.3	16.1	9.6	13.0	41.0	5
I get emotional support from my family	507	6.9	5.9	3.8	13.8	69.6†	0
I get support from my friends	500	15.6	10.6	10.2	16.0	47.6	7
My family has accepted my illness	499	3.0	3.4	4.2	10.4	79.0†	8
I am satisfied with family communication about my illness	507	4.3	3.8	5.1	12.2	74.6†	0
I feel close to my partner (or the person who is my main support)	488	16.6	4.9	4.1	9.8	64.6†	19
I am satisfied with my sex life	324	33.6	11.7	7.4	12.4	34.9	183

† , The subscale items for which more than 50% of participants indicated a ‘4’ (‘very much’).

The PWB subscale (refer to Table 4) found that the most common complaint (61.8%) was that they were forced to spend time in bed and the most common symptom experienced was nausea (53.4%). The PWB can be devastating for individuals dealing with cancer; however, EWB can also have a large impact on HRQoL. Nearly two-thirds of the participants in our study (65.9%) stated that they were ‘losing hope in their fight against their illness’ and were ‘worried about dying’ (56.8%). Despite these sentiments, 50.2% still felt ‘satisfied with how they were coping with their illness’. While experiencing challenges with PWB and EWB, 70.3% felt they had accepted their illness and 51.6% could enjoy life. Most participants felt well supported by their families (69%) and satisfied with family communication (74%) and 78.9% indicated that their family members had accepted their illness.

## Discussion

The sample of 507 participants was representative of the target population. The distribution of males versus females was 1:3, which was different from the approximately 1:1 distribution of females (51.3%) and males (48.6%) diagnosed with cancer in South Africa in 2018 (Stats SA 2023). The median age was 53 years, while the median age at diagnosis of cancer was 59 years for females and 64 years for males in 2018 (Stats SA 2023). The WHO (2022) determined that the most common types of cancer worldwide were breast, lung, colorectal, prostate and skin cancer, which is like our study but in contrast, cervical cancer was also prevalent.

The total FACT-G median score of a study conducted by Jeon et al. (2020) on individuals dealing with advanced non-small-cell lung cancer was 64. The aim of the study was to evaluate the prognostic value of HRQoL using the FACT-G. The total FACT-G median score was 76.7 in our study. This could be attributed to 47% of our participants not knowing what stage of cancer they have while the participants in Jeon et al. (2020) were all in the advanced stage. A similar study was conducted by Tchen et al. (2004) on patients dealing with breast cancer and found a total FACT-G median score of 77. The study of Tchen et al. (2004) matched their study participants with a healthy control group by age and the normative value for the HRQoL of the control group was 93. The study was conducted in Canada, which in 2023 was viewed as a high-income country while South Africa is an upper-middle income country (The World Bank 2024). No normative data for the HRQoL of the general population in South Africa utilising the FACT-G is available. The population of Tchen et al. (2004) only included breast cancer, which was also most prevalent in our study, although other types of cancers were also included. A study conducted by Chagani et al. (2017) in Pakistan (low-income country) assessed the HRQoL and its determinants in cancer patients undergoing chemotherapy. The mean for the overall HRQoL was 57.37, which was much lower than the overall HRQoL found in our study. It is interesting to notice that the SWB subscale score in the Chagani et al. (2017) was higher (22.3) than the score in our study (20.9), while all the other scores were lower. It is acknowledged that adverse effects are experienced during chemotherapy, which affects the HRQoL of individuals dealing with cancer and could thus explain the differences in findings (Chagani et al. 2017) as only 63.5% of the participants in our study were receiving chemotherapy.

The median value for the SWB in the study of Jeon et al. (2020) was 18.7 and in our study 22.2. Support from family, friends and partners positively influences HRQoL (Jabari et al. 2022) and effective coping strategies (Sari, Dewi & Dauley 2019) of individuals dealing with cancer. These results highlight the importance of support and are further substantiated by the findings of Jabari et al. (2022), indicating that this subscale showed the best scores on positively influencing an individual’s HRQoL. A qualitative exploratory study conducted in a hospital in Gauteng by Jansen van Rensburg et al. (2017) exploring the perception of individuals dealing with cancer found that the support from family, friends, their spouse and church members enhanced their HRQoL as the participants could share their experiences. Being separated from their family caused emotional distress as the participants were not only worried about their treatment but also their family. The philosophy of Ubuntu is promoted in African culture and is based on the concept that ‘a person is a person through other people’ (Taylor 2014) and includes ‘interdependency in terms of caring’ (Mabovula 2011) and sharing, which explains the negative experience of being separated from family and friends and the negative influence on HRQoL of individuals dealing with cancer. Social support is not only important in African cultures but also in other cultures.

The FWB of a patient directly impacts the quality of the patient’s emotional, physical and social well-being (Yeung et al. 2020). The median score of the FWB was 20 and in Jeon et al. (2020) 15. Almost three-quarters of participants felt that they had accepted their illness and just over half of the participants were happy with their lives. These results not only depict the positive influence FWB has on EWB, PWB and SWB and therefore on the HRQoL of the individual but also the influence of coping strategies as discussed earlier.

The median value for PWB in our study as well as in Jeon et al. (2020) was both 19. Most participants complained of nausea and that they were forced to spend time in bed. Pyszora et al. (2017) compared a group of individuals dealing with cancer in a palliative care department who participated in physiotherapy for 2 weeks, with a control group who received no physiotherapy. The therapy group reported a statistically significant reduction in their fatigue and drowsiness and rated their well-being higher than the control group. Eyigor (2010) found that individuals dealing with cancer who were bed-bound benefited from exercise. Individuals engaging in an exercise programme experienced an increase in their HRQoL, and cardiovascular capacity, as well as an improvement in their sleep and fatigue. Furthermore, a feeling of wellness developed, and their body mass index and immunity improved.

The median EBW subscale score was 18 and in Jeon et al. (2020) 15. In our study, half of the participants reported that they were coping well with their condition, but a larger percentage reported that they felt they were losing hope in their fight against their illness and were worried about dying. This corresponds with the research of Jabari et al. (2022) who found that participants also had the lowest scores for these items. Cancer is stressful and results in lifestyle changes. The process that takes place when an individual is dealing and coping with cancer is a unique experience and may be explained in terms of Lazarus and Folkman’s psychological stress model and the critical events model of Filipp, as cited in Homa, Ziarko and Litwiniuk (2023). According to these models, the source of stress and the consequences of the stress experiences arise not only from the physical aspects of the disease but also from past experiences, such as the patient’s assessment of the disease and their coping strategies. In the critical events model by Filipp, the concept of critical life events emphasises confronting stress by distinguishing the types of stresses, the so-called stressors of critical life events (CLEs). These stressors could be patients with no experience of a life-threatening disease or of their own experience or experience with a close family member. Hence, the differences in coping mechanisms and stress by individuals dealing with cancer will depend on their own or their family’s history of life-threatening disease (Homa et al. 2023).

### Clinical implications

Health-Related Quality of Life remains a complex concept, which is impacted by various individual factors both positive and negative. Our study indicates how the supportive role of healthcare professionals involving family, friends and partners as support during cancer treatment-related experiences influences HRQoL positively. The multi-disciplinary care of individuals dealing with cancer should be coordinated to address and evolve to ensure that the individual receives adequate and appropriate support to deal with the stress of the life-threatening disease and to maintain HRQoL despite the disease and the treatment-related experiences. Referral of individuals dealing with cancer to the appropriate healthcare professionals is essential to ensure the holistic well-being of the individual and their family. All other subscales of the FACT-G can also be positively addressed by physiotherapy interventions, which could be delivered individually or in group settings to improve the HRQoL of individuals dealing with cancer.

### Limitations

Patients were sampled from a single hospital at Universitas Annex in Bloemfontein. As such, the results may not be nationally representative, but the hospital is the referral hospital not only for the Free State but also for the Northern Cape and Lesotho. Participants who were unable to read, write or understand Afrikaans, English or Sesotho were excluded from our study, and because of urbanisation, there are also individuals dealing with cancer who are speaking other languages indigenous to the Free State.

### Recommendations

Similar studies should be conducted in other provinces in South Africa using the FACT-G questionnaire so that the results can be compared throughout the provinces and be more representative of the country. More specific HRQoL should be determined for each type of cancer as different challenges are experience depending on the type and stage of cancer that the individual is dealing with. These questionnaires are freely available online. Multiple linear regression analysis should be performed in future to explain the variability in the HRQoL, which would contribute to determining factors associated with HRQoL of individuals dealing with cancer.

## Conclusion

As one of the first studies determining the HRQoL of individuals dealing with cancer in the Free State using the FACT-G questionnaire, numerous findings deserve special attention. The findings demonstrated that the participants had the poorest results under the EWB subscale. Despite EWB being scored out of 24 rather than 28, these results still emphasise the need for improvements in psychosocial interventions to decrease the number of individuals experiencing challenges with EWB while dealing with cancer and treatment-related experiences. Therefore, it is crucial for physiotherapists to not only identify individuals with affected EWB but also to refer these individuals to other healthcare professionals. The results of the SWB indicated that HRQoL was positively influenced by SWB, highlighting the importance of a support structure. Functional Well-Being and PWB are crucial components of HRQoL in individuals dealing with cancer, and physiotherapists can play a valuable role in assisting with increasing the HRQoL of individuals dealing with cancer by providing exercise interventions, health education, support and referral where needed.
